# HealthRecSys: A semantic content-based recommender system to complement health videos

**DOI:** 10.1186/s12911-017-0431-7

**Published:** 2017-05-15

**Authors:** Carlos Luis Sanchez Bocanegra, Jose Luis Sevillano Ramos, Carlos Rizo, Anton Civit, Luis Fernandez-Luque

**Affiliations:** 1 Department of Architecture and Computer Technology Universidad de Sevilla, Seville, Spain; 2 eHealth Researcher, Toronto, Canada; 3 Qatar Computing Research Institute, Hamad Bin Khalifa University, Qatar Foundation, PO Box 5825, Doha, Qatar

**Keywords:** Patient Education, Health Recommender System, Natural Language Processing, Information Retrieval

## Abstract

**Background:**

The Internet, and its popularity, continues to grow at an unprecedented pace. Watching videos online is very popular; it is estimated that 500 h of video are uploaded onto YouTube, a video-sharing service, every minute and that, by 2019, video formats will comprise more than 80% of Internet traffic. Health-related videos are very popular on YouTube, but their quality is always a matter of concern. One approach to enhancing the quality of online videos is to provide additional educational health content, such as websites, to support health consumers. This study investigates the feasibility of building a content-based recommender system that links health consumers to reputable health educational websites from MedlinePlus for a given health video from YouTube.

**Methods:**

The dataset for this study includes a collection of health-related videos and their available metadata. Semantic technologies (such as SNOMED-CT and Bio-ontology) were used to recommend health websites from MedlinePlus. A total of 26 healths professionals participated in evaluating 253 recommended links for a total of 53 videos about general health, hypertension, or diabetes. The relevance of the recommended health websites from MedlinePlus to the videos was measured using information retrieval metrics such as the normalized discounted cumulative gain and precision at K.

**Results:**

The majority of websites recommended by our system for health videos were relevant, based on ratings by health professionals. The normalized discounted cumulative gain was between 46% and 90% for the different topics.

**Conclusions:**

Our study demonstrates the feasibility of using a semantic content-based recommender system to enrich YouTube health videos. Evaluation with end-users, in addition to healthcare professionals, will be required to identify the acceptance of these recommendations in a nonsimulated information-seeking context.

**Electronic supplementary material:**

The online version of this article (doi:10.1186/s12911-017-0431-7) contains supplementary material, which is available to authorized users.

## Background

Recent studies have shown an increasing trend in the use of the Internet as a search tool for health-related information [1–3]. Web 2.0 [4] allows contributions from any user in a network, which has given rise to a wealth of health-related information with a wide range of co-existing trustworthy sources [5, 6]. For this reason, screening tools can assist users in selecting relevant information.

Recommender systems are among the many solutions used to obtain valid information. When searching for an item, users obtain a list of recommended results that may match their preferences. Various filtering methods make it possible to refine and tailor these recommendations [7, 8]. Recommender systems can be divided into three basic groups: collaborative, context-based, and hybrid systems. Collaborative systems build on experience gathered from previous user experiences, i.e., items previously chosen by other users shape future results [9]. Context-based systems focus on the characteristics of an item, i.e., when searching for a camera, the recommendation output is based on its resolution, price, and color. Hybrid recommender systems combine features of context-based and collaborative systems [10]. Recommender systems can be also used to give additional item recommendations for a given item, such as the related videos that are shown by YouTube next to the user’s current video. These recommendations often relay user ratings, but can also be based on knowledge-based systems.

Recommender systems have been used in several applications for finding accurate information. They were introduced as a computer-based intelligent technique that assists people with the problem of information overload. These systems provide personalized solutions in various specific domains [11–13]. Recommender systems reflect the user’s interest and make proper personalized recommendation through several methods. Most current systems have adopted recently developed algorithms that use machine-learning [14–16], naive Bayes [16, 17], social-trust-based [18–21], constraint-based [22], case-based [23, 24], and matrix factorization [25, 26] approaches. Recommender systems are also found in clinical settings, mainly to assist health professionals, though some systems assist family members, patients, or caregivers [27–29].

Recent advancements in online recommender systems are enhanced by the “Semantic Web” [30], which allows for the extraction of vast amounts of information through metadata mining and artificial intelligence techniques [31]. Using these techniques, it is possible to rank and classify items based on terms that encompass several properties grouped into ontologies [32]. In the life sciences, ontologies play an important role in filtering relevant item and creating knowledge-based systems. Knowledge-based, cased-based, and social-trust-based approaches utilize user metadata, such as age and gender, to define recommendation rules. Machine-learning and naïve Bayes methods create models to learn users’ interests from their historical behavior. Matrix factorization learns a user’s latest interests by collaboratively factoring the rating matrix over historically recorded user-item preferences.

Health terms are also grouped into ontologies, creating an important potential resource for many applications, including recommender systems. Health ontologies usually have an application-programming interface (API) to precisely define their operation. One example of an API^1^ is Bio-ontology,^2^ which contains more than 600 health-related ontologies. Using Bio-ontology, Rivero-Rodriguez et al. recommended relevant links for a subset of health-related YouTube videos [33] by extracting corresponding clinical terms from the Medline Plus API for the International Health Terminology Standards Development Organization, which maintains SNOMED-CT, a multilingual clinical healthcare ontology.^3^

### Our previous work

**Fig. 1:**
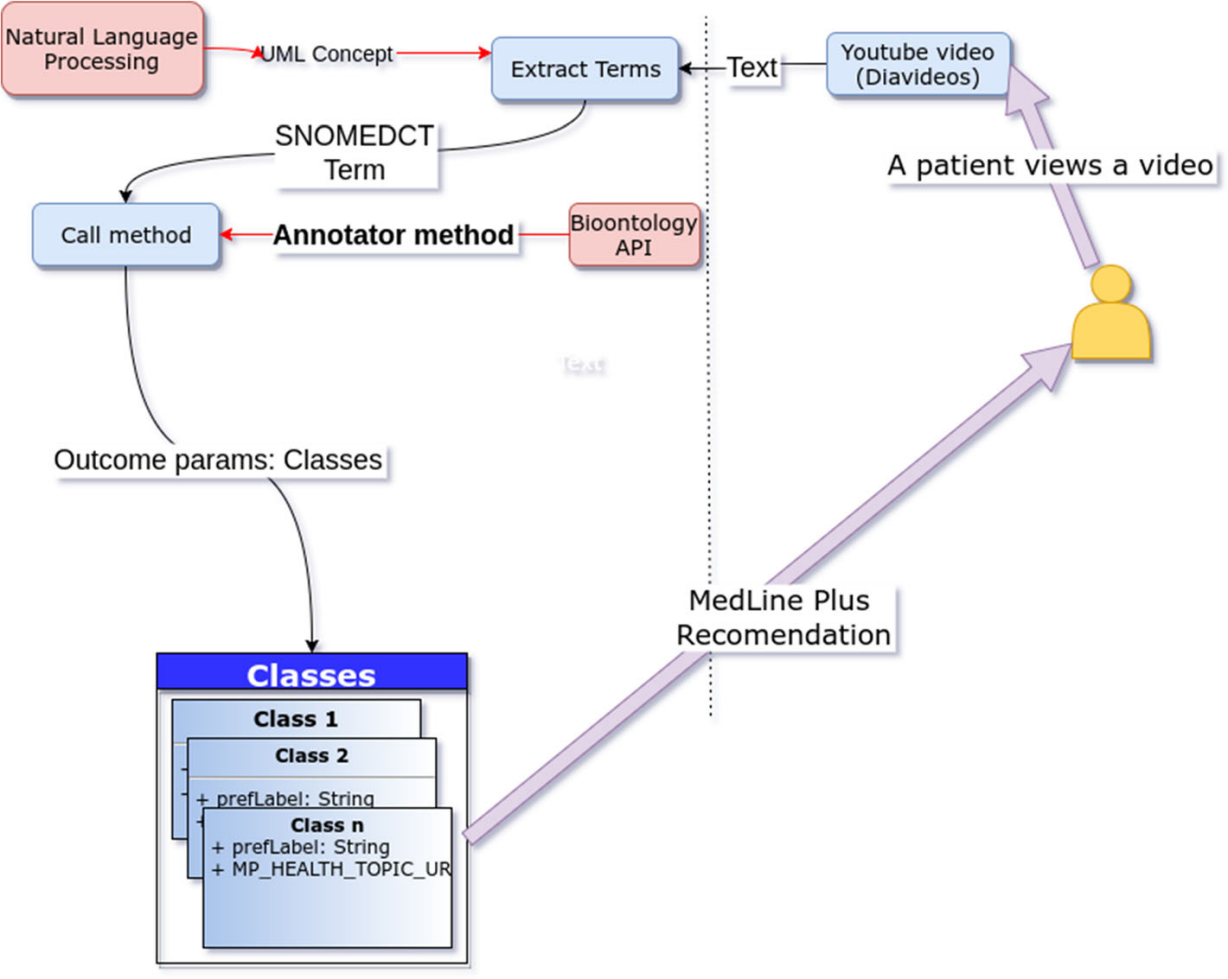
HealthRecSys Extraction of Medical Terms for Videos. Structure and logic of the extraction of medical terms and Medline Plus links for diabetes videos


This study is based on our previous work. Fernandez-Luque et al. reused algorithms from [33], but added the Bio-ontology API to improve the results for obtaining links from Medline Plus. In this study, we also rely on diabetes videos [34] for which we have already explored the use of semantic technologies to provide additional content recommendations [35]. Based on [33, 34], the proposed method gathers recommendations for Medline Plus links (see Fig. 1) from video subtitles to increase the number of associated terms using health ontologies. An additional movie file shows this in more detail [see Additional file 1]. An important limitation, both in the current and previous recommender systems, stems from the difficulty of mapping suitable terms to the ontology, especially when extracting representative terms from video content. One interesting approach to this problem uses natural language processing (NLP) [36–38] techniques, which can combine syntactic, semantic, and contextual analyses. NLP has previously been used in healthcare [39, 40], especially for mining electronic health records [41].

Additional file 1:HealthRecSys Study Overview. Video describing the HealthRecSys algorithm and the results of the study.


### Objectives

In online browsing, it is common to search for content related to online material currently being viewed. For example, after watching a video on YouTube, the watcher might look for additional content as part of an information seeking strategy. This search strategy has led to the creation of recommender systems that provide recommendations for related content. In this study, we explore the feasibility of recommending links to health educational content as a supplement to online health videos, focusing on recommendation methods that use semantic-based technologies to enhance online health content recommender systems. Further, this study investigates website recommendations that will enhance health videos, because video formats have shown the fastest growth on the Internet and it is estimated that, by 2019, video will constitute more than 80% of Internet traffic. 4

## Methods

In this study, we introduce HealthRecSys, a recommender system with Bio-ontology terms that generates Medline Plus links from text extracted from the metadata of selected YouTube videos (see Fig. 1).

Our recommender system involves several steps: A) collecting filtered words from the title of a video, B) collecting any one SNOMED-CT term from the title, C) collecting a group of SNOMED-CT terms from the title, and D) determining the union of the results of steps B and C. Step A uses a “stop word” filtering system (i.e., that avoids preposition, adverbs, and similar terms), and steps B and C are combined with SNOMED-CT to extract web links from MedlinePlus.

### Algorithm design

**Fig. 2:**
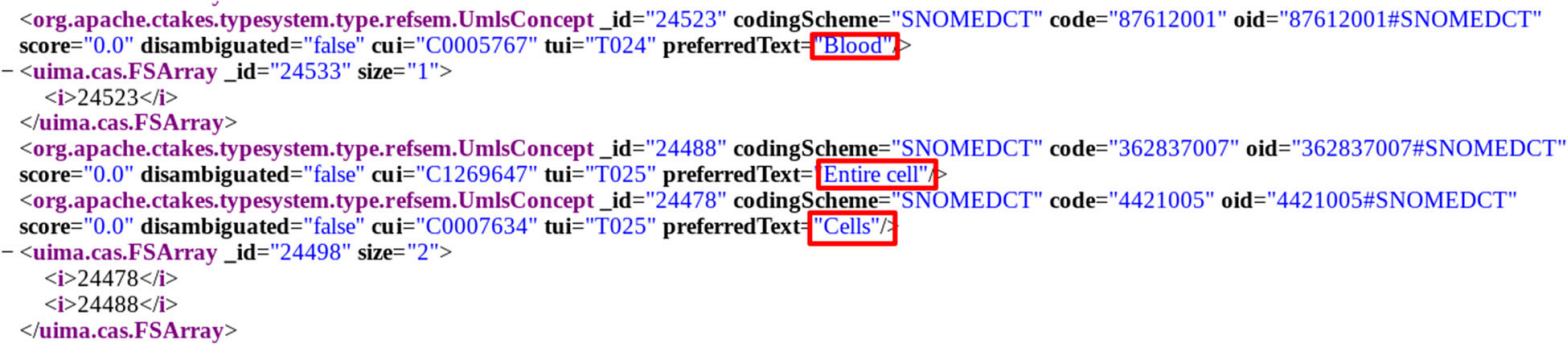
cTakes XML Example with Video Metadata. Example of XML source code from the cTakes result for a video related to blood cells

We selected keywords (or terms) from video metadata (i.e., the video title, description, and subtitles). These keywords are used to identify semantic terms from Medline Plus. Fig. 1 shows the **Term Extraction** process for diabetes videos from YouTube. The algorithm contains two steps:

- **Source term collection**: the video title, description, and subtitle are collected as possible terms.
- **NLP:** In this case, NLP is applied to the title description and video subtitles using the cTAKES framework.^5^ This is a health-specific NLP implementation that extracts SNOMED-CT health terms from text. See Fig. 2 for an example of extracted metada of a video.

We conducted a text analysis using the Unified Medical Language System (UMLS)^6^ with SNOMED-CT annotations to match the cTAKES framework. To achieve this, we inject the original video metadata files (with title, description, and subtitle) procedures from the UMLS library, resulting in an XML file that contains a morphological, syntactic, and semantic analysis.

From this file, we filtered the *UmlConcept* labels that contain collected terms from the SNOMED-CT ontology properties. For instance, Fig. 2 shows example XML for the terms *Blood*, *Entire Cell*, and *Cells*. The cTAKES configuration uses the standard pipeline *AggregatePlaintextFastUMLSProcessor* to extract the SNOMED-CT terms.

**Fig. 3:**
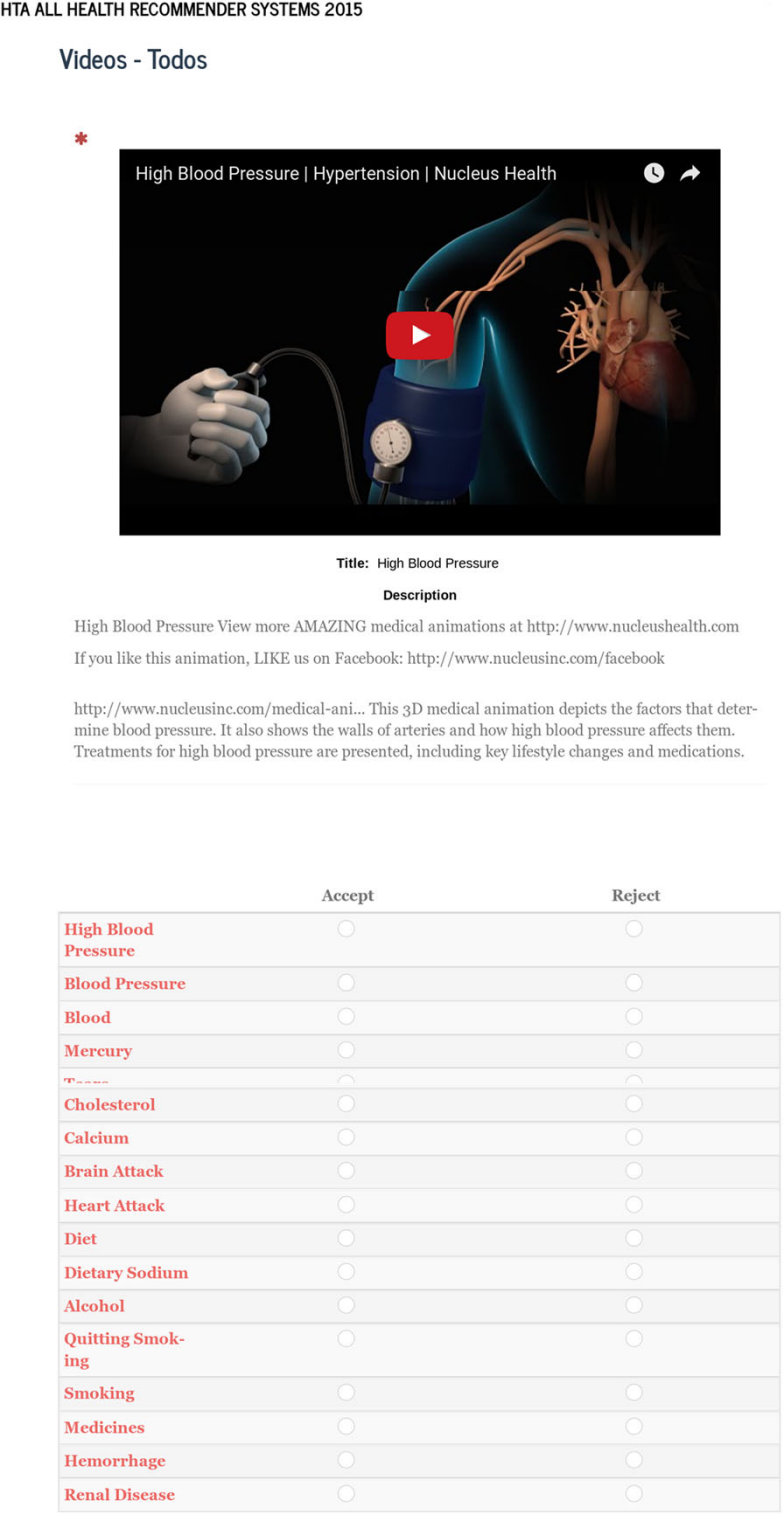
Web Form for Raters. Example screenshot of the video and rating system presented to raters. (Video source: https://www.youtube.com/watch?v=diG519dFVNs)

To work with the UMLS library, we used a profile license.^7^ Appendix 1 shows the configuration used to run the cTAKES execution.

Once the SNOMED-CT terms are extracted, we cross-match them with the terms from the Bio-ontology API to find synonymous MedlinePlus terms. These outputs allow us to obtain a web link from the *MP_HEALTH_TOPIC_URL* MedlinePlus property, which is obtained via a Representational state transfer (REST) endpoint from the associated extracted term, which allows us to provide trusted recommendations to end users. For instance, the example terms *Blood* and *Stem Cell* both have corresponding Medline Plus links,^8^^.^ 9

Given that the number of SNOMED-CT vocabulary terms is larger than those on MedlinePlus, we anticipated that many results would not have matching terms. Although Bio-ontology offers an Annotator Web service that annotates user-provided text (e.g., journal abstracts) with relevant ontology concepts, this feature was not used for this work.

For practical reasons, we ignored isolated terms from SNOMED-CT that did not have a Medline Plus match. Although it is possible to select other ontologies to find a corresponding Medline Plus term, in this paper, we focus on results obtained only with these two ontologies.

### Datasets of videos and raters

**Fig. 4:**
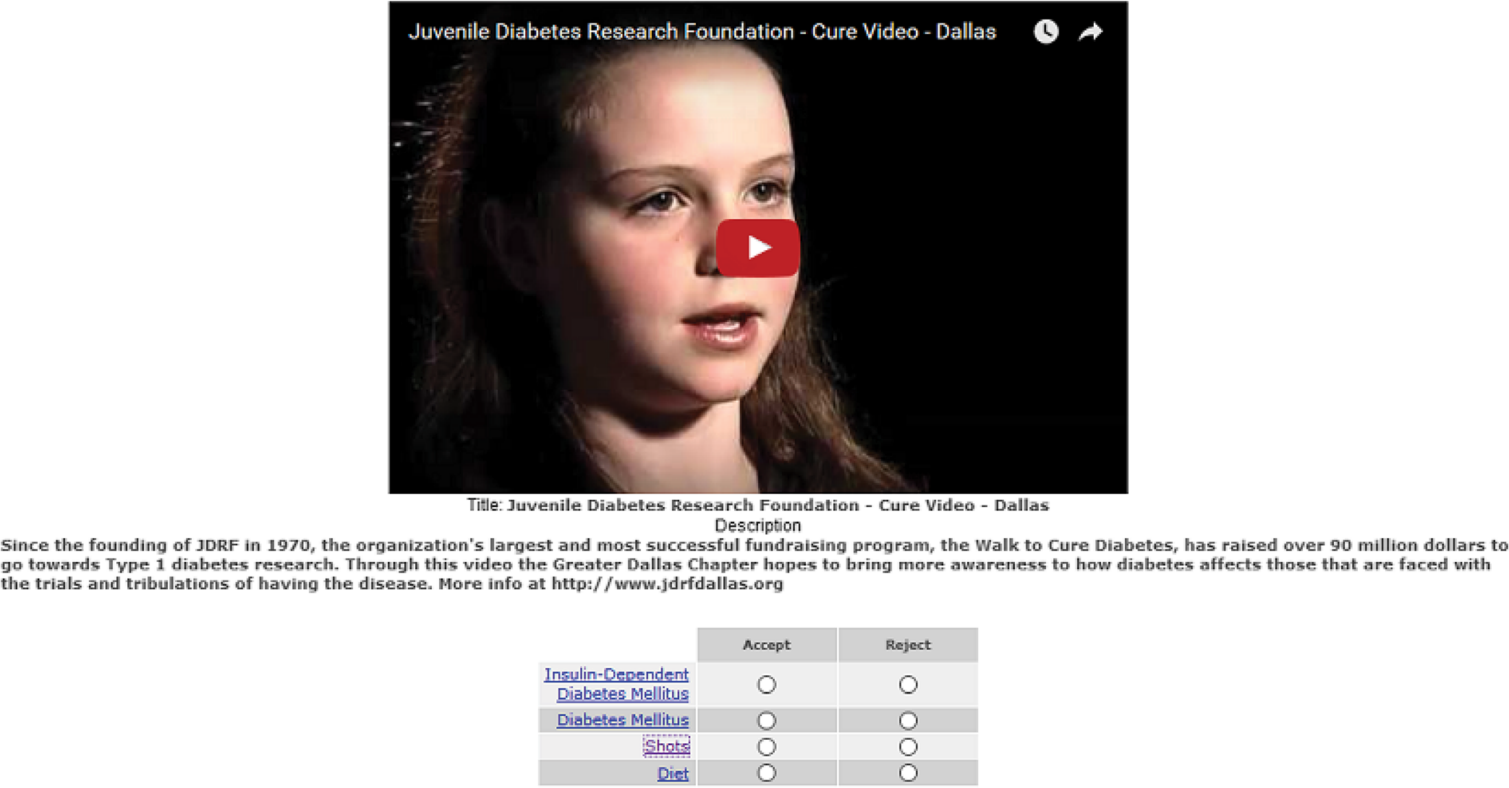
Juvenile Diabetes Research Foundation Video. Example diabetes video from the Diabetes Research Foundation and links extracted from MedlinePlus. (Video source: https://www.youtube.com/watch?v=i7ft-6vR-Ic)

We assigned 26 health professionals (raters) to the three set of videos divided by topic (general medicine, diabetes, or hypertension). We recruited these healthcare professionals directly either by email or other means, based on their familiarity with health topics and online health. After explaining to them the goals of the project and acquiring informed consent, the raters were asked to determine if the recommended links for a given video were relevant for the video topic. The exercise of rating the recommendations was not based on any personal information from the participants, but rather their expert opinion of a web tool (see Figs. 3 and 4). As such, this research does not involve human subjects (the study does not obtain information about living individuals).

Our dataset contained 53 videos, some of which had been utilized in our previous research [33]: a) 10 general medical videos (i.e., general health-related videos extracted from hospital YouTube channels), b) 22 videos about diabetes, and c) 21 videos about hypertension.

To rate the relevance of the videos and recommended links, we used Cohen’s kappa to determine the level of agreement between two given raters. Kappa is defined as follows [42]:

1 $$ k=\frac{Pr(a)- Pr(e)}{1- Pr(e)}, $$

where *Pr(a)* is the relative observed agreement and *Pr(e)* is the hypothetical chance of agreement. Therefore, this formula calculates the ratio of observed agreement to hypothetical agreement by chance. If the raters are in complete agreement, then *k = 1*. A *k* coefficient greater than 0.80. indicates good agreement for a given recommendation.

Cohen's kappa was calculated using the *irr* package of the R application (version 3.3.1 on linux-gnu). The method in question is *kappa2(ratings, “unweighted”)*. This function includes the vector of the rater values.

For each category of videos (general medical, diabetes, and hypertension), we selected a pair of reviewers with a high level of inter-rater agreement, based on Cohen’s kappa, to have consistent rater agreement. The pair of raters had a Cohen’s kappa inter-rater agreement of 0.626 for the general medical videos (*z* = 4.35, *p*-value = 1.33 × 10^−05^), 0.582 for diabetes (*z* = 6.47, *p*-value = 9.9 × 10^−11^), and 0.717 for hypertension (*z* = 7.7, *p*-value = 1.31 × 10^−14^).

In the next step, we selected videos and links with an acceptable level of inter-rater agreement based on the Cohen’s kappa values. Using the algorithm described in the previous section, we generated 510 recommended MedlinePlus links, but evaluated only the first five recommendations for each video, as our recommender system limits the number of recommendations. The final dataset contained 10 general medical videos with 48 recommended links, 22 diabetes videos with 102 recommended links, and 21 hypertension videos with 103 recommended links.

This approach allowed us to focus the evaluation on videos and links for which there was a homogenous agreement level among professionals. The rationale of this approach is relayed in our previous research, which highlighted the lack of consensus between professionals on certain types of health videos [43].

### Evaluation metrics of the recommendations

We used two metrics to evaluate the relevance of the recommended links for a given video. These metrics, precision at *k* [44] and normalized discounted cumulative gain [45], are widely used to evaluate search algorithms in information retrieval and indicate the relevance of the “top” retrieved results. The importance of focusing on the top retrieved results is based on the web browsing behavior of users, as they tend to focus only on the top few item suggestions.

### Precision at *k*

Precision (also called positive predictive value) is the fraction of retrieved instances that are relevant, in our case, this is the relevance of the links recommended for a given video. Precision is calculated as

2 $$ \mathrm{Precision}=\frac{\left|\mathrm{Trusted}\ \mathrm{Recommendations}\left|\ {\displaystyle \cap}\right|\mathrm{Recovered}\ \mathrm{Recommendations}\right|}{\left|\mathrm{Recovered}\ \mathrm{Recommendations}\right|}. $$

The precision at *k* (*P*@*k*) [46, 47] accounts for the order of the returned recommendations and is calculated as the fraction of the first *k* accepted links to all *k* links.

### Normalized Discounted Cumulative Gain

The normalized discounted cumulative gain (nDCG) is another common information retrieval metric [45]. It is a measure of ranking quality, where DCG_k_ are highly relevant documents appearing lower in a search result and the ideal discounted cumulative gain (iDCG_k_) is the DCG of the vector with all links with an accepted value:

3 $$ n D C{G}_k = \frac{DC{G}_k}{iDC{G}_k}\ . $$

## Results

To evaluate each recommendation, we considered two scenarios: a) robust and b) moderate. In the robust scenario, we consider as relevant only those link recommendations that are supported by both raters. In the moderate scenario, we consider a link to be relevant if at least one rater agreed with the recommendation. The moderate case is most appropriate when the risk of misinformation is low, while the robust scenario is the most appropriate when there is greater potential to spread misinformation.

In these scenarios, P@k and nDCG_k_ were calculated as follows. The relevance of the *k* first link recommendations is calculated as follows for each recommended link *j* (*1 ≤ j ≤ k*):

(a) If both raters approve link *j*, it is accepted (its value is 1, or *relevant*).

(b) If both raters do not approve link *j*, it is rejected (its value is 0, or *irrelevant*).

(c) In the case in which one rater approves link *j* and the other rejects it, in the robust scenario, link *j* is considered irrelevant (value 0), whereas in the moderate scenario, it is considered relevant (value 1).

The P@k results are shown in Table 1 and nDCG results are showed in Table 2. Overall, the performance of the recommender system was higher when giving recommendations for the general medicine and diabetes videos.

**Table 1:** Mean precision @ K recommended links

	Mean Precision@k (robust case)			Mean Precision@k (moderate case)		
	*k* = 3	*k* = 4	*k* = 5	*k* = 3	*k* = 4	*k* = 5
General Medicine	0.77	0.65	0.5	0.87	0.80	0.70
Diabetes	0.71	0.71	0.68	0.89	0.85	0.81
HTN	0.48	0.45	0.39	0.62	0.57	0.53

The evaluation based on nDCG (see Table 2) shows similar patterns, and lower performance when recommending links for hypertension videos. As expected, the relevance of the links decreased with an increase in the number of recommended links for a given video (*k* = 5)

**Table 2:** Mean nDCG for K recommended links

	Mean nDCG_k_ (robust case)			Mean nDCG_k_ (moderate case)		
	*k* = 3	*k* = 4	*k* = 5	*k* = 3	*k* = 4	*k* = 5
General Medicine	0.78	0.7	0.5	0.88	0.83	0.75
Diabetes	0.73	0.74	0.72	0.90	0.87	0.85
HTN	0.51	0.49	0.46	0.65	0.61	0.58

## Discussion

The results show that it is feasible to recommend relevant links for health videos using a semantic-based recommender system. However, there are several concerns that deserve special attention. Although positive overall, recommendation performance varied across the different topics used in this study, which could be due several factors. For example, there might be fewer links related to diabetes than other topics (e.g., hypertension), thus limiting the potential items that can be recommended. Further, our semantic-based approach might also suffer from the semantic-gap between the layperson’s language and a medical thesaurus. Although work has been done to develop a Consumer Health Vocabulary, this has not been implemented in our approach; additionally, the semantic gap may differ across health topics [48].

In contrast, our approach of using semantics to identify relevant links allows the algorithms to find links that are related to synonyms and disambiguation. Still, this poses some additional challenges. For example, in a video titled *Juvenile Diabetes Research Foundation − Cure Video – Dalas,* 10 our algorithm extracted the term “shots,” which resulted in a recommendation for a link regarding the importance of vaccination (a topic of relative importance in diabetes). One advantage of relying on medical terms is that our algorithm has an enhanced capability to reduce the number of links that have no relation to the video content, which is an important limitation of previous studies, where such terms could not be avoided [33, 34].

Recommender systems can play a major role, not only in education, but also in supporting behavioral changes for a wide range of health conditions [49–51], including smoking cessation [51]. In such cases, the recommendations are not only chosen with regard to content, but also with respect to timing, and consider different psychological health factors (aka user context) [52]. Our work does not address context-awareness regarding the time and place of the recommendations. However, by providing trustworthy recommendations for websites when a user is watching a video, we can support complex health information seeking [53, 54].

The applications of recommender systems in the health domain are still emerging. Therefore, we lack common evaluation methods that can allow us to compare work across separate studies in this topic [29, 55]. There are examples in the literature of recommender systems in the health domain that, for example, provide recommendations based on a personal health record [56]. In our case, we deal with a very different type of content-based recommendation, as we are not recommending content for a given user but rather for a given health educational item.

Our work is aligned with previous studies in which health information is enriched with additional content [56]. There is still quite a substantial knowledge gap on how people search for online health information, and, even more importantly, on how that affects the health behaviors of the information seeker [57]. Our recommender system approach does not aim to provide recommendations personalized for a user, but rather to provide further reliable information for users watching a health video. This content-based recommendation approach is crucial for supporting the current patterns of health consumers looking for multiple sources when searching for health information online [58].

Most previous studies of health recommender systems do not address their impact on health outcomes; in contrast, we do so using information retrieval accuracy metrics. This approach has the potential to create risks for health consumers, which is one of our motivations for using health professionals in this evaluation. Ekstrand et al. recently reviewed potential ways in which health recommender systems can do harm and the ways to minimize potential harm [59]. Giving wrong or potentially misleading health information can be a cause for serious concern; for example, recently, the FDA forced the company 23andMe to remove and edit personalized health information regarding genetic health risks [60]. Further, health information can be used for unhealthy purposes (e.g., the abuse of diuretics for weight loss is common in people with eating disorders).

### Limitations

Our study relies on the ratings of hundreds of recommended links for given videos. However, these ratings were given by healthcare professionals and not health consumers. As explained in our previous work, professionals and consumers often disagree on the relevance of health content [43]. Experiments with health consumers will be required to further evaluate recommendation quality.

Note that our study only investigates the feasibility of this approach. Consequently, extrapolating the results to larger studies is necessary. Ideally, further studies will consider more users (and not necessarily healthcare professionals). In addition, our rating approach was rather simplistic, considering the multiple quality dimensions of health videos [35]. Further, the ideal evaluation should take place in a real information seeking scenario and not a simulated one because many factors affect information seeking by health consumers, including stress or literacy levels [53]. The patient perspective was not explored in this study because we consider it to be more ethically appropriate to first study the feasibility of an approach with health experts. Patients’ perspectives and acceptance can also vary substantially across age, health literacy levels, and other factors. Future research will need to explore the application of our method in a patient portal with additional content and users.

Another limitation of our study is that our video dataset is not generalizable. We selected several topics of high importance (diabetes and hypertension), but we cannot extrapolate that our approach will work with other health topics. A major challenge to generalizing semantic-based approaches such as ours is the gap between medical and consumer health vocabularies [61]. Because we use content generated by health organizations (not individuals) and a medical ontology, we might expect more difficulties when recommending links to consumer-generated content.

## Conclusions

This study demonstrated that a semantic-based recommender algorithm can provide relevant education health websites as further reading for a given health video. The relevance of websites recommended by our system decreased as we provided more recommendations, but HealthRecSys still performed well with up to five recommended links per video. Because user browsing behavior is often limited to a few items, this does not pose a serious limitation. Conversely, our approach can reduce the burden of health consumers when searching for reliable additional health educational content. Further, the speed of navigation to a reliable source, as identified by Strauss, is an important factor in information seeking [62].

Future improvements to recommender systems will incorporate more semantic analytics and perhaps be able to determine the patient’s context (i.e., mood) to make better recommendations. It will be possible to use this algorithm to recommend content and videos to counterbalance misinformation, find information on controversial topics, and filter out videos with little scientific acceptance. For instance, a video that promotes steroid consumption could recommend information alerting the individual to their potential negative effects.

## Abbreviations

API: Application-programming interface
NLP: Natural language processing
UMLS: Unified Medical Language System

## Footnotes

1 Bio-ontology API endpoint documentation http://data.bioontology.org/documentation

2 Bio-ontology website http://www.bioontology.org/

3 SNOMED-CT website http://www.ihtsdo.org/snomed-ct

4
http://www.cisco.com/c/en/us/solutions/collateral/service-provider/visual-networking-index-vni/complete-white-paper-c11-481360.html

5 cTAKES website http://ctakes.apache.org/

6 UMLS website https://www.nlm.nih.gov/research/umls/

7 UMLS web license profile https://uts.nlm.nih.giv//uts.htmlprofile

8 Blood: https://www.nlm.nih.gov/medlineplus/blood.html

9 Cell: https://www.nlm.nih.gov/medlineplus/stemcells.html

10 Juvenile Diabetes Research Foundation https://www.youtube.com/watch?v=i7ft-6vR-Ic
